# Supplementary material for: Transcriptome Analysis Reveals Vimentin-Induced Disruption of Cell–Cell Associations Augments Breast Cancer Cell Migration
Source: Cells. 2022 Dec 13;11(24):4035. doi: 10.3390/cells11244035 (PMC9776984; doi:10.3390/cells11244035)
Supplement: Supplementary file 1 [file cells-11-04035-s001.zip › DEGs and enrichment analysis.zip!/DEGs and enrichment analysis/WT_VIMvsVector GO enrichment/DOWN/WT_VIMvsVector_down.CCbar.pdf]

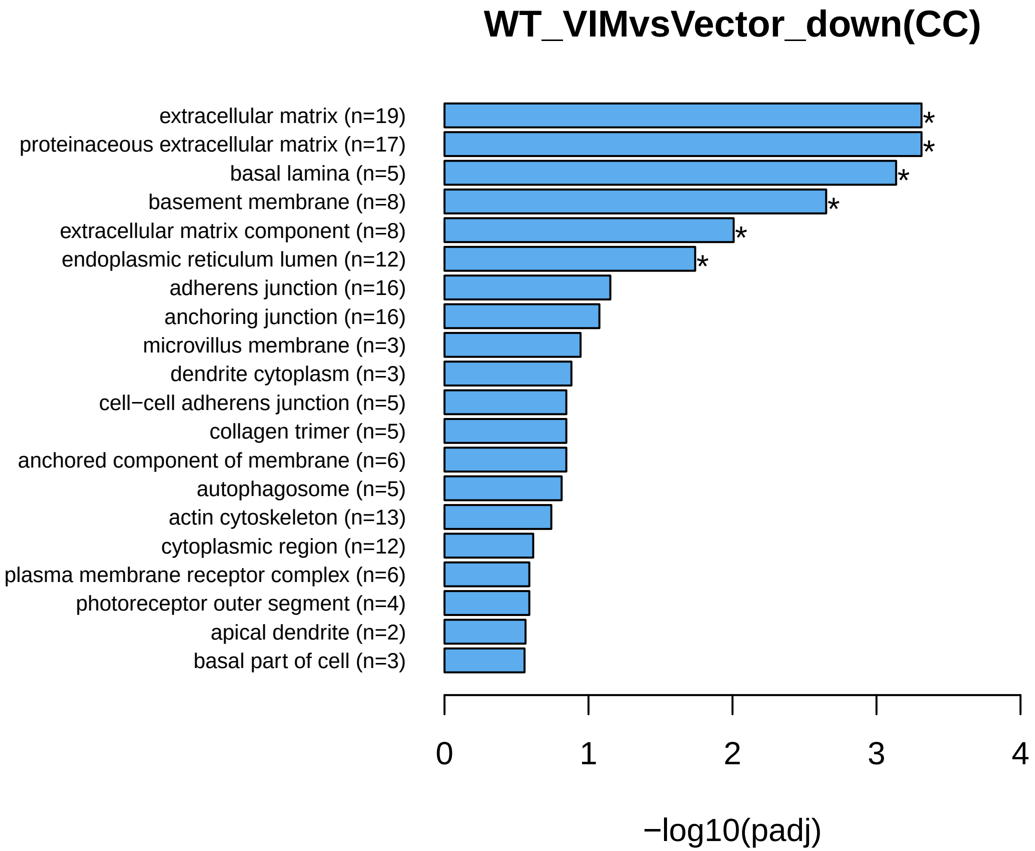
WT_VIMvsVector_down(CC)
extracellular matrix (n=19)
proteinaceous extracellular matrix (n=17)
basal lamina (n=5)
basement membrane (n=8)
extracellular matrix component (n=8)
endoplasmic reticulum lumen (n=12)
adherens junction (n=16)
anchoring junction (n=16)
microvillus membrane (n=3)
dendrite cytoplasm (n=3)
cell−cell adherens junction (n=5)
collagen trimer (n=5)
anchored component of membrane (n=6)
autophagosome (n=5)
actin cytoskeleton (n=13)
cytoplasmic region (n=12)
plasma membrane receptor complex (n=6)
photoreceptor outer segment (n=4)
apical dendrite (n=2)
basal part of cell (n=3)
0
1
2
3
4
−log10(padj)
